# Assessing the harmonization of structured electronic health record data to reference terminologies and data completeness through data provenance

**DOI:** 10.1002/lrh2.10468

**Published:** 2024-10-21

**Authors:** Keith Marsolo, Lesley Curtis, Laura Qualls, Jennifer Xu, Yinghong Zhang, Thomas Phillips, C. Larry Hill, Gretchen Sanders, Judith C. Maro, Daniel Kiernan, Christine Draper, Kevin Coughlin, Sarah K. Dutcher, José J. Hernández‐Muñoz, Monique Falconer

**Affiliations:** ^1^ Department of Population Health Sciences Duke University School of Medicine Durham North Carolina USA; ^2^ Duke Clinical Research Institute Duke University School of Medicine Durham North Carolina USA; ^3^ Department of Pediatrics Children's Hospital of Philadelphia Philadelphia Pennsylvania USA; ^4^ Department of Population Medicine Harvard Medical School Boston Massachusetts USA; ^5^ Department of Population Medicine Harvard Pilgrim Health Care Institute Boston Massachusetts USA; ^6^ US Food and Drug Administration Silver Spring Maryland USA

**Keywords:** common data models, data provenance, data quality, harmonization

## Abstract

**Introduction:**

(1) Assess the harmonization of structured electronic health record data (laboratory results and medications) to reference terminologies and characterize the severity of issues. (2) Identify issues of data completeness by comparing complementary data domains, stratifying by time, care setting, and provenance.

**Methods:**

Queries were distributed to 3 Data Partners (DP). Using harmonization queries, we examined the top 200 laboratory results and medications by volume, identifying outliers and computing summary statistics. The completeness queries looked at 4 conditions of interest and related clinical concepts. Counts were generated for each condition, stratified by year, encounter type, and provenance. We analyzed trends over time within and across DPs.

**Results:**

We found that the median number of codes associated with a given laboratory/medication name (and vice versa) generally met expectations, though there were DP‐specific issues that resulted in outliers. In addition, there were drastic differences in the percentage of patients with a given concept depending on provenance.

**Conclusions:**

The harmonization queries surfaced several mapping errors, as well as issues with overly specific codes and records with “null” codes. The completeness queries demonstrated having access to multiple types of data provenance provides more robust results compared with any single provenance type. Harmonization errors between source data and reference terminologies may not be widespread but do exist within CDMs, affecting tens of thousands or even millions of records. Provenance information can help identify potential completeness issues with EHR data, but only if it is represented in the CDM and then populated by DPs.

## 1 INTRODUCTION

High‐quality data are a key component of an evidence generation system that leverages clinical enterprise to support the learning cycle of knowledge generation, dissemination, and uptake.^1^ In the context of real‐world data (RWD), assessing data quality often means ensuring that a dataset is suitable for a given analysis or investigation (i.e., fit‐for‐purpose).^2^, ^3^ RWD often goes through several transformation and cleaning steps as they move from raw source to a final analytical dataset.^3^, ^4^ The ability to track and document these steps is critical in the review and interpretation of evidence generated from RWD as well as promoting reproducibility and transparency.^5^ There have been several efforts to standardize this process, but they are largely focused on the activities that occur between the initial dataset used for the research analysis (e.g., data formatted into a Common Data Model [CDM]) and the final result.^6^, ^7^ Far less attention has been paid to the transformation and cleaning steps that occur between source systems and the CDM.

Understanding these transformations is important because electronic health record (EHR) data are not natively captured, generated, or stored according to reference terminologies or interoperability standards.^8^, ^9^ Before RWD can be used for multi‐center research or learning health system activities, raw source data must be *harmonized* to a common representation.^10^, ^11^, ^12^, ^13^, ^14^, ^15^, ^16^ Often this includes converting local representations to standard reference terminologies.^17^, ^18^ For laboratory results, this means mapping to Logical Observation Identifiers Names and Codes (LOINC©), a terminology for representing laboratory tests, and for medications, mapping to RxNorm, a reference terminology for medications.

There are several reasons why the mapping process remains a challenge. Within the average health system, 100–150 laboratory tests may make up 85% of the total volume, but the remaining records may consist of hundreds or thousands of unique tests.^19^ The methods used within a test may have changed over time, meaning that different codes may need to be assigned based on specific date ranges. In addition, certain metadata may be missing or unavailable (e.g., specimen source), particularly for historical results. Finally, while there are tools to assist analysts in the mapping process (e.g., RELMA),^20^ the analysts performing the mapping may not be subject matter experts. Medications have similar challenges. The average health system may have tens of thousands of unique medication concepts once all the different combinations of ingredient, dose, and dose form are taken into consideration.^21^, ^22^ Within RxNorm, the same medication concept can be represented at different levels of granularity. To find the appropriate code for a given medication, analysts either need to independently query the RxNorm reference database or rely on cross‐walks that are part of the medication databases within the EHR (e.g., Medi‐Span, Multum, First Data Bank). These cross‐walks are proprietary to the vendor and seldom validated externally. Table S1 shows examples of potential mapping issues.

There is existing work related to RWD quality,^9^, ^12^, ^23^, ^24^, ^25^, ^26^, ^27^, ^28^, ^29^ particularly within distributed research networks, which have been developed to support multi‐center RWD studies at scale. Examples include PCORnet®, Sentinel, the Health Care Systems Research Network (HCSRN), the Observational Health Data Science Initiative (OHDSI), and topic‐specific networks.^15^, ^30^, ^31^, ^32^, ^33^, ^34^, ^35^ In these networks, participating organizations (or Data Partners [DP]) harmonize data from their source system(s) into a CDM and then execute data curation procedures to characterize data quality.^36^, ^37^, ^38^, ^39^, ^40^ These foundational procedures can be supplemented with study‐specific data quality investigations.^41^, ^42^ Data curation procedures can only indirectly determine if a record was appropriately mapped to a CDM value set or reference terminology, however. For instance, PCORnet maintains a check that examines whether the specimen source of a CDM laboratory record is consistent with the specimen source associated with the corresponding LOINC code (i.e., records for a urinalysis have a specimen source of “urine”).^43^ A mismatch might indicate a potential problem with the record but does not provide enough information to determine the cause without directly accessing the source data.

**FIGURE 1:**
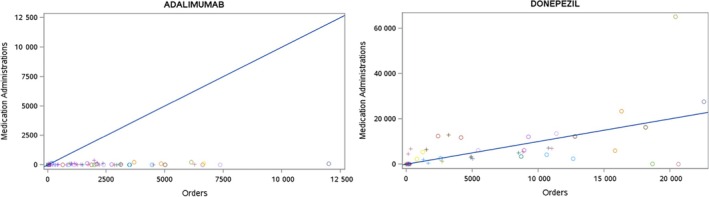
Ratio of medication administrations versus orders for adalimumab (left) and donepezil (right) for data partners within PCORnet. Each dot represents the ratio for a data partner; the blue line represents perfect concordance between medication administrations and orders. For adalimumab, the ratio for all data partners is close to zero, suggesting no medication administration data associated with this medication. For donepezil, the ratio is clustered closer to the 1:1 line, though there is an extreme outlier in the top right corner. Dots far above the line suggest missing medication orders data.

Beyond source data comparisons, another potential strategy for assessing data transformations is to consider data domains that are overlapping and/or complementary to one another. Within the EHR, for instance, structured information about medications can be found in multiple forms, including prescriptions (orders), dispensed medications, billed procedures, and inpatient, outpatient or home health medication administrations. The presence or absence of a given medication across these domains, or even the variability in counts/rates from one domain to another, can help determine if there are any underlying issues with the quality or completeness of the medication data (see Figure 1). Other EHR domains like diagnoses and procedures do not have quite as many representations as medications, but comparisons are still possible based on the workflow used to create the record (e.g., clinician‐entered, generated during the billing process). In this manner, we can use the provenance, or origin of the data from within the EHR, to help investigate potential data quality issues.

## 2 QUESTIONS OF INTEREST

**FIGURE 2:**
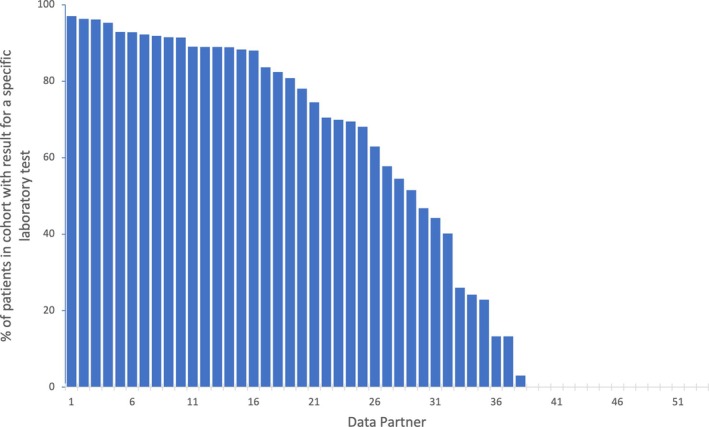
Percentage of patients in a cohort with a laboratory result. Each bar represents the results from a data partner. The “empty” or 0% values on the right represent data partners who have likely not loaded the relevant results into their Common Data Model. For the data partners in the ~3%–65% range, it is unclear whether their results reflect missing data or just practice variation.

This work focused on two main objectives. The first is on *data harmonization*, specifically the process of mapping raw structured EHR data to a common format and reference terminology. We assess the harmonization of laboratory results and medication orders versus medication administrations (inpatient and outpatient) and characterize the severity of issues or discrepancies that are uncovered. The second objective focuses on *data completeness* (using the definition from Kahn), or presence or absence of data.^25^ While the completeness checks for CDMs are focused mostly on whether fields in a record are present or absent, we are trying to assess whether “all” records for a given concept available in source system(s) are captured in a CDM (e.g., procedures for x‐ray mammograms). Some networks have developed quality checks that look for the total absence of a concept,^40^ but trying to determine the inverse, whether that concept is “complete” is more difficult, as shown in Figure 2. To assess completeness and whether data may be missing, resulting in undercounting, we developed queries to compare complementary data domains, looking at profiles of records across time, care setting, and provenance, for a series of conditions. In this manner, we seek to extend the capabilities of foundational, network‐level data quality assessments, which provide broad, population‐level characterization, without having to conduct a full‐blown study‐specific quality assessment that investigates specific variables for a defined population and time period.

## 3 METHODS

### 3.1 Data partners

Three DPs participated in this project: Mayo Clinic, University of Florida, and Duke University. All participating DPs are members of PCORnet^32^, ^44^ and have harmonized their data into the PCORnet® CDM. Table S2 provides information on the terminologies used to represent data within the PCORnet® CDM. Queries were written to execute against the PCORnet CDM^45^, ^46^ but are generalizable to any CDM that contains the relevant fields and metadata. Each DP's PCORnet® CDM contains EHR data from all the hospitals and clinics of their academic medical center and affiliated health system. All DPs currently use Epic as their enterprise EHR. When reporting results, the identities of each DP are masked and reported as DP1, DP2, and DP3 (the numbering does not correspond to the ordering defined above).

For queries related to diagnoses and procedures, we stratify results by provenance, or origin within the EHR. In the PCORnet® CDM, there are 4 non‐null provenance types—clinician‐entered (also referred to as “ordered”), EHR billing (“billed”), claims, or derived (e.g., derived through natural language processing). EHR billing data come from health system financial systems and are analogous to the information that would be found on an administrative claim from a health plan. Table S3 includes information on the availability of diagnoses and procedure records by provenance for the 3 DPs, as well as for PCORnet as a whole.

### 3.2 Harmonization queries

To assess the harmonization of structured laboratory results and medication orders and medication administrations, we ran several analyses. We computed summary statistics (minimum, median, maximum) on the number of LOINC/RxNorm codes that were associated with each “raw” or non‐harmonized lab/medication name. We also characterized the number of names that were associated with each LOINC/RxNorm code. We generated a list records having LOINC/RxNorm codes mapped to more than one name or vice versa, and names without a corresponding LOINC/RxNorm code, or vice versa. We also assessed the fidelity of each mapping, comparing the ingredient associated with the assigned RxNorm code with that of the medication name and the description of the LOINC code (i.e., the test name) with the raw laboratory name. For records that were mismatched, we characterized the severity of the discrepancy using the categorization found in Table 1. Each harmonization query was limited to the top 200 raw names by volume (record count) within each data domain. The query selected the top raw names by volume and then extracted all records within that table that contained that name. The top 200 names could differ across DPs, though we expected overlap.

**TABLE 1:** Example of issues that can be uncovered through the mapping assessment and their overall severity (i.e., potential impact on studies).

Severity	Example issue	Rationale
Critical	Lab test mismatch (incorrect LOINC code) Multi‐ingredient drug uses single‐ingredient RxNorm code Single‐ingredient drug uses multi‐ingredient RxNorm code	(1–3) The LOINC/RxNorm codes that are assigned to these records are incorrect and would not actually represent the test result or exposure to the specified medication.
Major	Ingredient‐level RxNorm code utilized when more specific code is available (single‐ingredient drugs only) More granular RxNorm code used than supported by the data	The ingredient is correct, but the other metadata is missing, meaning those records may be excluded if the drug has forms that are not part of an analysis (i.e., topical creams). This example is the inverse—records that should have been excluded were included.
Moderate	Generic medication uses brand name RxNorm code Brand name medication uses a generic‐level RxNorm code Laboratory tests measuring different components of the same analyte share the same LOINC code	Any study that looking for the use of a specific brand of medication will include extra records. Studies that are looking at the use of a specific branded medication will miss records. Test may be overly specific for the given code, and results should not necessarily be grouped with others.
Minor	Distribution of lab results is an outlier for a given LOINC. Point‐of‐care lab tests assigned the same code as those run in the clinical lab	The test may be only used on specific populations (e.g., inpatients), which may bias results. While the tests may report results on the same component, they should generally not be assigned the same LOINC code. Any studies that are specifically targeting the testing method will not be possible.
Unknown	Medication/laboratory test name is uninformative	Cannot determine if record was correctly mapped based on the information in the name field.

*Note*: The higher the severity, the greater the percentage of studies that would be affected. Note that even if an issue is deemed to be lower severity, it can be considered as critical if a study has to rely on the property in question.

Abbreviation: LOINC, Logical Observation Identifiers Names and Codes.

### 3.3 Completeness queries

For the completeness queries, we selected 4 conditions of interest: chronic hepatitis C (Hepatitis C), chronic kidney disease (CKD), chronic obstructive pulmonary disease (COPD), and pulmonary arterial hypertension (PAH). For each condition, we defined a set of clinical “concepts” within the categories of diagnoses, procedures, medications, and laboratory tests to characterize that population. Each concept can be represented in the PCORnet® CDM by one or more codes and was selected due to its expected association with each condition. We included up to 8 concepts in each category, though additional concepts were included if the relevant code lists had been defined in prior work. The conditions, categories, and their associated concepts are shown in Table 2. The conditions were selected based on scientific interest and the research expertise of the authors and other colleagues at FDA. We also tried to ensure that we included conditions that would be present in a variety of care settings and would capture different populations to improve the generalizability of our findings.

**TABLE 2:** Conditions and associated concepts used in the completeness queries.

Condition	Categories and concepts
Chronic hepatitis C	**Diagnoses:** Hepatic decomposition, hepatitis B, human immunodeficiency virus (HIV) **Medications:** Interferon, nucleoside analogue, non‐structural protein 5a inhibitor **Procedures:** Liver ultrasonography, liver biopsy, hepatitis A/B vaccination **Labs:** Albumin (serum), bilirubin, hepatitis C virus ribonucleic acid, international normalized ratio
Chronic kidney disease	**Diagnoses:** Anemia, dyslipidemia, hyperkalemia, metabolic acidosis, pericarditis, thyroid dysfunction, uremic bleeding, uremic neuropathy **Medications:** Erythropoiesis‐stimulating agents, iron, angiotensin‐converting enzyme inhibitors, angiotensin receptor blockers, beta blockers, loop diuretics, amlodipine, sodium/glucose cotransporter‐2 inhibitors **Procedures:** Abdominal imaging (ultrasound, computerized tomography [CT], magnetic resonance imaging, x‐ray), dialysis, renal arteriography/venography, voiding cystourethrography pyelography, kidney biopsy **Labs:** Albumin (serum, urine), blood urea nitrogen (BUN), creatinine (serum, urine), estimated glomerular filtration rate, ferritin, hematocrit (HCT), hemoglobin, parathyroid hormone, protein (urine)
Chronic obstructive pulmonary disease	**Diagnoses:** Asthma, congestive heart failure, coronary artery disease, depression, diabetes, environmental exposure, lung cancer, osteoporosis, pulmonary heart disease, sleep disorders **Medications:** Beta adrenergic agonists, muscarinic antagonists, oral/inhaled glucocorticoids, oxygen supplementation, first line antibiotics, macrolides, fluoroquinolones, antipseudomonal penicillin, cephalosporins, aminoglycoside **Procedures:** Chest X‐ray, chest CT, pulmonary function test, pulse oximetry, ventilation **Labs:** Alpha‐1 antitrypsin, partial pressure of oxygen, partial pressure of carbon dioxide, acidity, bicarbonate, hemoglobin, leukocytes, platelets, sputum gram stain, and culture
Pulmonary arterial hypertension	**Diagnoses:** Congenital heart disease, HIV, rheumatoid arthritis, scleroderma, systemic lupus erythematosus **Medications:** Prostacyclins, endothelin receptor antagonists, soluble guanylate cyclase stimulator, phosphodiesterase type 5 inhibitors **Procedures:** Echocardiogram, electrocardiogram, cardiac catheterization, ventilation perfusion scan **Labs:** Alkaline phosphatase, alanine transaminase, Bilirubin, B‐type natriuretic peptide, BUN, carbon dioxide, calcium, chloride, creatinine (serum), glucose, HCT, human chorionic gonadotropin, platelets, potassium, sodium, thyrotropin

A query was developed and distributed to DPs to generate patient counts for each condition and category/concept, stratified by year (2016–2021), encounter type (e.g., outpatient, inpatient, emergency department, other), and provenance (e.g., clinician‐entered/order, EHR billing). We conducted exploratory analyses looking at trends over time within a given DP and across DPs by provenance and encounter type, and at differences in the rates of provenance within a category (e.g., provenance for diagnoses within a condition). Due to space constraints, the results of the encounter type and laboratory result trends are not shown.

## 4 RESULTS

### 4.1 Harmonization

The results of the data harmonization queries are summarized in Tables 3 and 4. Table 3 provides the median, minimum, and maximum number of codes associated with each raw name and vice versa, for the ordered and administered medication records at each DP, as well as laboratory tests. All laboratory tests can be represented with a single LOINC code, and the PCORnet® CDM provides guidance that DPs should map their medications to RxNorm codes that can fully represent the associated ingredient in a single code, so the expected number of associated records for each is 1 (there may be instances where an inpatient mixture is associated with more than one, as we discuss below). The median value is 1 for DPs 1 and 2. With DP3, the median value for medication order codes associated with a given raw name and laboratory result raw names associated with a given code is 2. We also see larger maximum values in all three DPs. Reasons for this include the use of general names for mixture medications and the use of ingredients as the medication name. Additional explanations for the medication values at DP1 (administrations) and DP3 (orders) are provided in Table 4 below. The larger maximum values for laboratory tests can occur when DPs receive data from multiple hospitals that may utilize a single LOINC code for tests with slightly different raw names (e.g., WBC and White Blood Cells), or that use LOINC codes with differing levels of granularity for the same test (which may or may not be a data quality issue).

**TABLE 3:** Number of codes associated with a given “raw” (non‐harmonized) name and number of raw names associated with a given code (median, minimum, and maximum) for medication orders, administrations, and laboratory tests (assumption is median ~ 1).

		DP1	DP2	DP3
Number of codes associated with a given “raw” name	Medication orders	1 (1, 2)	1 (0, 1)	2 (1, 6287)
	Medication administrations	1 (1, 805)	1 (1, 3)	1 (1, 4)*
	Laboratory results	1 (1, 4)	1 (1, 2)	1 (1, 4)
Number of “raw” names associated with a given code	Medication orders	1 (1, 5)	1 (1, 11)	1 (1, 5)
	Medication administrations	1 (1, 46)	1 (1, 12)	1 (1, 1)*
	Laboratory results	1 (1, 4)	1 (1,4)	2 (1, 8)

*Note*: For this analysis, if two raw names represented the same general concept but were represented slightly differently (e.g., GLUCOSE [METER] and GLUCOSE [POINT‐OF‐CARE]), they were treated as different raw names.

* For DP3, the raw names in the administration table are integers, not text strings.

**TABLE 4:** Examples of discrepancies seen between the raw name and name associated with the assigned code during harmonization.

Type	Site	Raw name	Name associated with code(s)	Notes
Medication administrations	DP1	Bivalirudin pediatric infusion	Bivalirudin 250 mg injection; sodium chloride 9 mg/mL injectable solution	Single raw name (mixture medication) is associated with multiple codes. Almost all of DP1's top administration records are mixtures and are affected by this issue.
	DP1	IV builder peds continuous; NICU LVP infusion builder; TPN adult continuous; TPN adult cyclic; TPN neonatal continuous; TPN peds continuous	Magnesium sulfate	Multiple raw names associated with a single ingredient. All are mixture medications with common ingredients. Almost all of DP1's top administration records are mixtures and are affected by this issue.
	DP1	ZZ IMS template	~800 unique codes	Generic template name. Unable to assess mapping.
	DP2	Phenylephrine (PF) 1 mg/10 mL (100 mcg/mL) in 0.9% NACL IV syringe	Medicone hemorrhoidal	Mapping error, affecting ~50 K records and ~25 K patients (<1% of total). Critical issue.
	DP2	Prenatal vitamin with calcium No. 72‐IRON 27 mg‐folic acid 1 mg tablet	Ferrous fumarate, folic acid, iron	Multi‐ingredient medication listed as multiple records. No records for vitamin/calcium, so technically an error or critical issue. Affects ~85 K records and ~45 K patients (<1% of total).
	DP2	PF 1 mg/10 mL (100 mcg/mL) in 0.9% NACL IV syringe; lisinopril 5 mg tablet	Little noses decongestant; lisinopril 5 mg oral tablet (Zestril)	Raw medication is a generic, while RxNorm code indicates a specific brand for that generic medication. Most of DP2's administration records exhibit this issue. Moderate issue.
	DP3	<Integer>	<RxNorm description>	Raw names represented as integer. Unable to assess mapping. Affects all of DP3's administration data.
Medication orders	DP2	11 different raw names	<NULL>	11 different raw names are not associated with a code, affecting ~2.4 M records and ~1 M patients (roughly 5% of the total analyzed). Critical issue.
	DP3	Oxycodone	Acetaminophen 325 mg/oxycodone hydrochloride 2.5 mg oral tablet	Single‐ingredient raw name and multi‐ingredient code. There were 16 instances of this at DP3, though only ~2 K records and 1 K patients. Major issue.
	DP3	Heparin	Lidocaine 20 mg/mL topical cream	Mapping error between raw name and code. Critical issue. At DP3, there were ~70 such instances, affecting ~500 K records and ~250 K patients (<1% of total orders).
	DP3	Heparin; heparin flush	Multiple heparin records	Heparin and Heparin flush are different formulations, but DP3 has the same RxNorm codes assigned to both Heparin and Heparin Flush raw names. Could be considered a critical issues. Affects ~5.5 M records and ~1 M patients.
	DP3	Non‐formulary medication	~6000 different codes	Uninformative raw name. Unable to assess mapping.
Laboratory results	All DPs	Glucose Lvl; glucose, random, S; glucose, POCT; glucose, POCT, WB	Glucose (mass/volume) in blood	Point‐of‐care lab associated with same LOINC as one run in clinical lab; relatively minor error, unless analysis depends on testing method. Affects millions of records/patients across all DPs (<1% of total).
	All DPs	EOS ABS; EOS EOS; EOS PCT AUTO	Eosinophils (#/volume) in blood by automated count Eosinophils/100 leukocytes in blood by automated count	Several labs that are part of the complete blood count panel report results as counts and %. In this case, the same raw name is associated with both types of results. One is likely mapped incorrectly. Critical issue. This issue affects tens of thousands of records/patients across all DPs (<1% of total).
	DP1	Bilirubin direct	CD3 + CD4+ (T4 helper) cells (#/volume) in blood	Mapping error (affecting ~6 K records/patients, <1% of total). Critical issue.
	DP1	Hematocrit	Hemoglobin S/hemoglobin. total in blood	
	DP1	BNP; NP PRO BNP	Natriuretic peptide B prohormone N‐terminal (mass/volume) in serum or plasma	Two raw names use the same LOINC code. May be overly specific for BNP records. Could be considered a moderate issue.
	DP2	Chloride	Creatinine (mass/volume) in serum or plasma	Mapping error; affects ~266 K records and ~50 K patients (<1% of total). Critical issue.
	DP3	K	Clot formation (Time) in blood by thromboelastography	Mapping error; affects <5 K records/patients (<1% of total). Critical issue.

Abbreviations: DP, Data Partners; LOINC, Logical Observation Identifiers Names and Codes.

Table 4 provides examples of the types of discrepancies seen in the data, the magnitude of the issue, and potential implications. Some of the examples are considered minor, as in the case where point‐of‐care laboratory tests are mapped to the same LOINC as those run in a clinical lab. More serious are laboratory tests mapped to an incorrect LOINC code and medication raw names that are associated with the wrong code or no code at all. For DP3, raw names for medication orders are generally stored at the ingredient level (e.g., morphine) instead of a string that incorporates the ingredient, strength, and dose form (e.g., morphine sulfate 40 mg tablet), which means a single name may be associated with dozens of codes, though the name with the greatest number of codes at DP3 was a non‐informative “non‐formulary medication.” For DP1, there was a generic name for administration of inpatient mixtures (“ZZ IMS TEMPLATE”), which was associated with 805 codes. The use of non‐specific terms to represent some medication administrations at DP1 and medication orders and administrations at DP3 also made it difficult assess the underlying mappings.

### 4.2 Completeness

**FIGURE 3:**
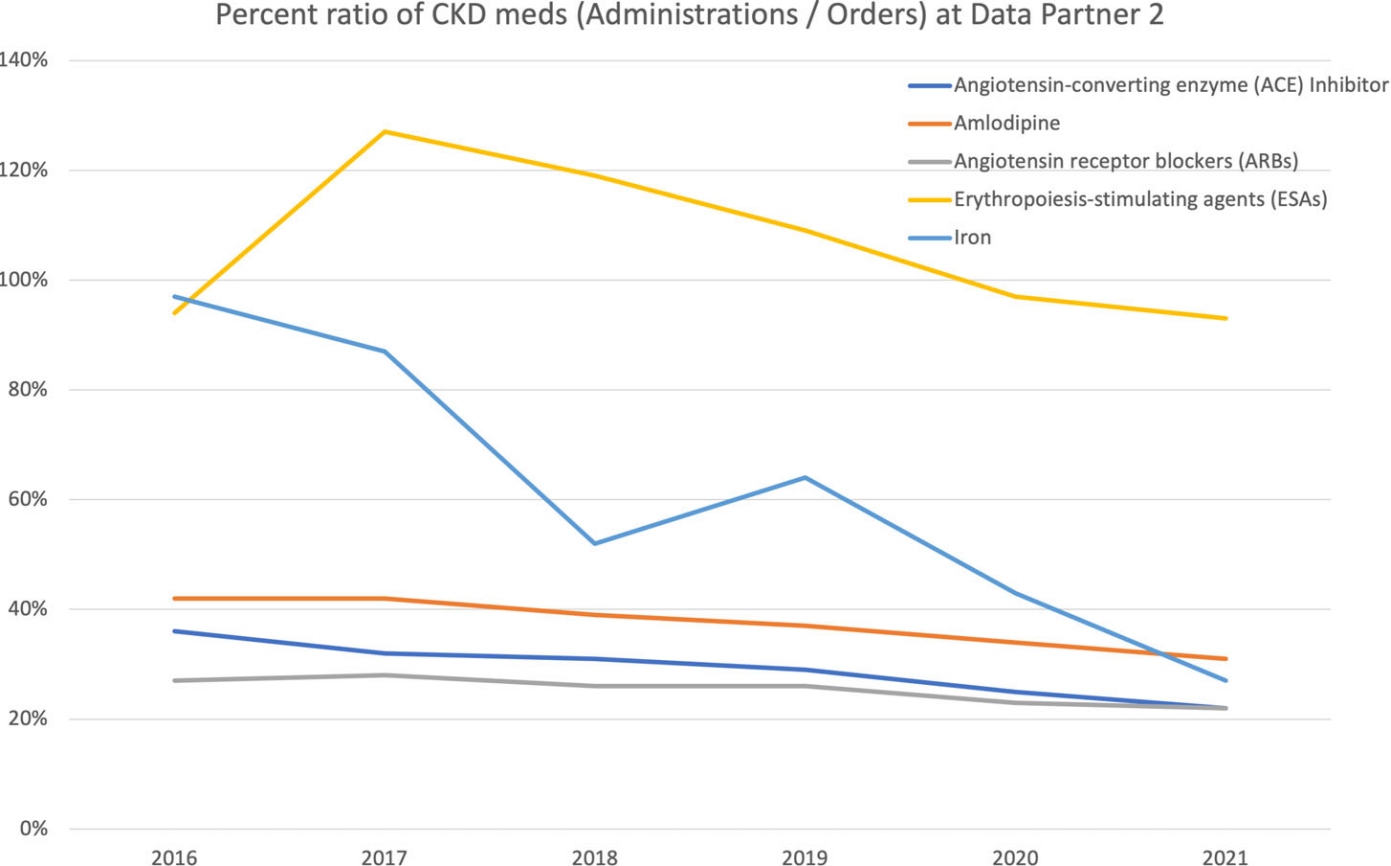
Percent ratio of medications (administrations/orders) for the chronic kidney disease (CKD) cohort by year at Data Partners 2.

**FIGURE 4:**
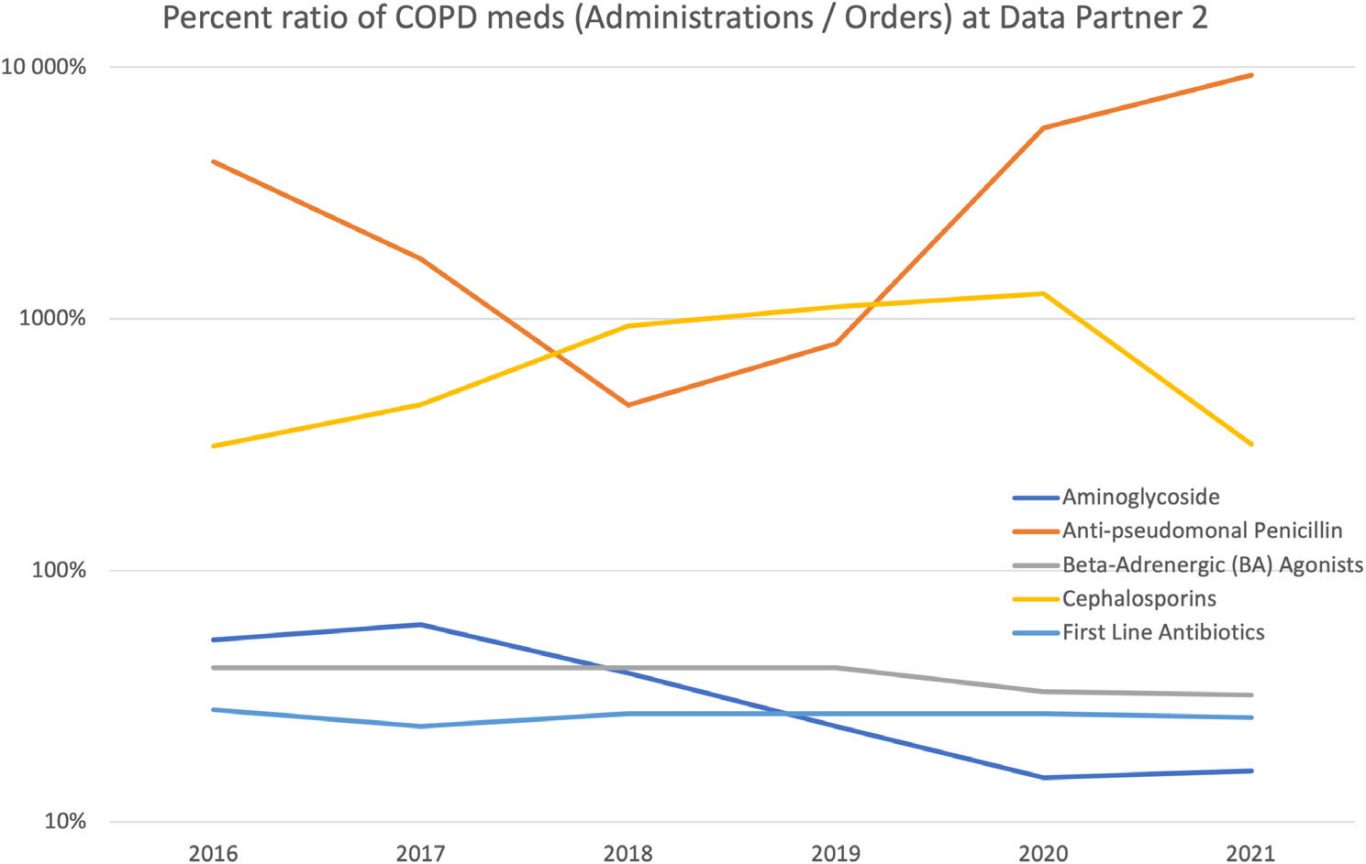
Percent ratio of medications (administrations/orders) for the chronic obstructive pulmonary disease (COPD) cohort by year at Data Partners 2. Note that the Y‐axis is represented in a log scale.

Table 5 and Figures 3 and 4 provide illustrative examples of the results from the data completeness queries. Table 5 shows the percentage of patients in the CKD cohort who have records for diagnosis and procedure concepts, broken down by data provenance (CKD diagnoses at DP1, CKD procedures at DP2). The proportion of patients with a given concept who have billed diagnoses at DP1 is >90% for each concept, while the proportion who have clinician‐entered diagnoses ranges from ~3% to ~77% (noting that some patients have diagnoses from both). For DP2, the proportion of patients with a procedure‐based concept who have billed procedures ranges from 71% to 99%, while the proportion who have clinician‐entered/ordered procedures ranges between ~50% and ~87%.

**TABLE 5:** Different rates of completeness for CKD concepts by provenance for DP1 (diagnosis) and DP2 (procedure).

	2016		2017		2018		2019		2020		2021	
	*N*	%	*N*	%	*N*	%	*N*	%	*N*	%	*N*	%
Chronic kidney disease diagnosis concepts at data partner 1												
**Number of unique patients**	10 279		10 974		11 896		12 614		12 674		13 007	
**Anemia**	5210		5690		6283		6582		6609		6999	
Clinician‐entered/ordered	3292	63%	3935	69%	4299	68%	4446	68%	4409	67%	4660	67%
Billing	4934	95%	5397	95%	5973	95%	6329	96%	6287	95%	6713	96%
**Pericarditis**	394		420		464		526		613		651	
Clinician‐entered/ordered	83	21%	83	20%	91	20%	105	20%	82	13%	125	19%
Billing	388	99%	418	100%	458	99%	522	99%	607	99%	643	99%
**Uremic bleeding**	778		901		858		887		809		828	
Clinician‐entered/ordered	597	77%	693	77%	664	77%	680	77%	584	72%	594	72%
Billing	707	91%	804	89%	787	92%	810	91%	750	93%	762	92%
**Metabolic acidosis**	987		1207		1276		1596		1783		1868	
Clinician‐entered/ordered	37	4%	41	3%	43	3%	41	3%	59	3%	64	3%
Billing	973	99%	1192	99%	1260	99%	1573	99%	1760	99%	1845	99%
Chronic kidney disease procedure concepts at data partner 2												
**Number of unique patients**	12 297		13 558		14 617		16 626		16 600		20 772	
**Biopsy, kidney**	145		156		140		171		157		199	
Clinician‐entered/ordered	91	63%	101	65%	73	52%	92	54%	79	50%	110	55%
Billing	123	85%	131	84%	121	86%	145	85%	135	86%	172	86%
**Dialysis**	1249		1295		1257		1409		1377		1437	
Clinician‐entered/ordered	1071	86%	1071	83%	1063	84%	1207	86%	1198	87%	1224	85%
Billing	1241	99%	1290	99%	1251	99%	1396	99%	1370	99%	1422	99%
**Pyelography**	240		275		283		296		306		405	
Clinician‐entered/ordered	182	76%	218	79%	230	81%	229	77%	230	75%	322	79%
Billing	192	80%	215	78%	202	71%	231	78%	230	75%	302	75%

*Note*: Patients with each concept (e.g., Anemia) have an associated diagnosis or procedure record. Results are then stratified by provenance (i.e., clinician‐entered/ordered or billing).

Abbreviation: CKD, chronic kidney disease.

Figures 3 and 4 provide an indirect assessment of completeness for the medication concepts by illustrating the ratio between administration and ordered records for CKD and COPD medication concepts at DP2. While not all medications that are ordered will be administered, we expect clinical workflows to remain the same and therefore the ratio to remain stable over time, unless clinical practice changes and a given medication (or class of medications) is used more or less frequently, or in different care settings (e.g., first dose of a medication is no longer administered in the clinic but taken by the patient at home). For instance, with medications used in CKD patients (Figure 3), the ratio of iron administrations to orders declines drastically over time, from close to 100% in 2016 to ~25% in 2021. When looking at COPD medications (Figure 4), we see examples of extreme administrations/orders ratios (~300% to 9000%). Both of these are indicative of data quality issues (see Section 5).

## 5 DISCUSSION

Analyses that rely on RWD sources, including those related to pharmacoepidemiology and learning health system activities, fundamentally assume that the code (e.g., ICD, CPT, RxNorm, LOINC) assigned to a record is correct. While CDM‐based data quality checks can determine whether a record is conformant to a given specification or that the values generally fit within a given range or distribution, any check would not be able to determine whether a record with a given code (e.g., RxNorm code of 993 770, acetaminophen 300 mg/codeine phosphate 15 mg Oral Tablet) was correctly assigned without looking at the source data. Similarly, failing to take the provenance, or origin, of the data into account means an analysis may have an incomplete view of the care delivered at a given institution. In the past, this has been somewhat mitigated by the fact that RWD analyses often directly involve the institutions who are contributing data and who can investigate data issues. As the number of de‐identified RWD repositories grows, along with the desire to create large, aggregated datasets to support activities like artificial intelligence, the ability for contributing health systems to assess data quality issues becomes more limited. This clearly demonstrates the need for methods that can assess the harmonization and completeness of data within CDMs or other RWD sources.

Within the harmonization results, among the data mappings that we reviewed, we identified several issues that could impact analyses. For instance, there were cases where the selected RxNorm code for an ordered medication specified more information than was available in the raw medication name field (e.g., name specified an ingredient, while the RxNorm code defined a brand name), though there were also cases where the raw medication name appears to have been improperly assigned (e.g., all raw medication names are a generic ingredient). This may be reflective of how the underlying PCORnet® CDM was populated but also reflects how some data sources may not be suitable for certain analyses without providing additional context as to how the medication codes are assigned. There were also instances where records with a non‐null medication name was not mapped to an RxNorm code (i.e., null RxNorm code), meaning that those records are “invisible” for analytical purposes. We also identified a handful of medications that appeared to be true mapping errors, and many examples where a single inpatient mixture medication within the EHR was represented by multiple PCORnet® CDM records. While it appeared that all relevant ingredients were present in the PCORnet® CDM, study teams would need to ensure that those records are indeed populated to avoid an incomplete mapping of the source EHR record. It is worth noting that given that we conducted our analysis on the top 200 medications by volume, any single issue could impact thousands or tens of thousands of records within a database. In addition, it is possible that additional harmonization issues could occur more frequently with less common medications.

With laboratory results, we found only a handful of mapping errors that involved a complete mismatch between LOINC and lab name. As with medications, this low number of errors would still affect thousands or tens of thousands of records (albeit a small fraction of the total). There were also several “minor” mapping issues that would affects millions of records at each DP (e.g., point‐of‐care tests sharing the same LOINC as those run in the clinical lab). These issues would not necessarily impact an analysis that was agnostic to the testing method but would have implications for any analysis or decision support tool that took the method into account. These findings are consistent with results reported elsewhere.^47^

For the completeness queries, we found that stratifying results by provenance yielded insights into the underlying quality of the data. For many diagnosis concepts at DP1, we saw much lower rates of completeness for records of clinician‐entered/ordered provenance compared with EHR billing records. We assume that these are not deliberate instances of “upcoding,” where patients are fraudulently made to look as if they have more serious conditions than warranted, but rather represent diagnosis codes that are assigned during the medical coding process by incorporating other information from the patient's chart. The level of completeness for EHR billing was generally quite high (>95%), though there were a handful of concepts with rates in the 88%–90% range. If only clinician‐entered diagnoses are available for a study, there could be significant missingness, at least compared to what appears in billing data. With procedures, the trends were more mixed in DP2. It should be noted that DP2 was the only DP that populated both ordered and billed procedures, making it difficult to assess completeness in the other DPs. Many procedures had low rates of completeness in DP2's EHR billing data. This might be due to the billed procedures being captured in an ancillary financial system whose data are not loaded into the PCORnet® CDM, because procedures were ordered but completed at an external facility, or in rare cases, because a procedure was ordered but never completed (the guidance for the PCORnet® CDM is to only include completed procedures). For diagnoses and procedures, having access to both types of data provenance provides the best coverage.

For medications, comparing the number of those that are administered to prescribed orders can be also informative. For a given medication, this ratio should be relatively stable over time. Large changes may indicate missing records, though they may also be reflective of changes in practice (e.g., less use of medication), or practice patterns (e.g., first dose of a medication no longer administered during a clinic visit, resulting in fewer administration records), which is a potential limitation of the measure. Extreme ratios may also be indicative of missing records. With DP2, ratios >1000% for some COPD medications are likely due to missing medication order records (e.g., data not loaded into the PCORnet® CDM, different codes used to represent a medication as an order and as an administration) and are an illustration of the potential value of these comparisons to identify data quality issues.

### 5.1 Limitations

This study was designed to highlight the potential benefits of assessing data by provenance, and we did see differences across the various diagnosis and procedure concepts. However, within this work, we lack a gold standard comparator (e.g., claims), so we do not know whether the absence of data in one stream of provenance represents missing data or some type of error; this is true of EHR‐based datasets in general. This is most evident with procedures, with the low rates of completion for billing data. It is unlikely that a procedure would be conducted without a bill being generated since procedures are costly to the healthcare system, so assuming the records represent completed procedures (and not those ordered but denied by insurance), a billing record should exist somewhere. Linking these data to claims and providing a third type of data provenance would allow for cross‐checking to determine if the ordered procedure was simply completed at an external facility.

In addition, our work primarily focused on the potential “harms” of missing data, which would result in undercounting in an analysis. Another harm comes from overcounting, when the same event is represented differently across different data streams within the same health system and counted multiple times (e.g., a single medication order can result in multiple medication administrations). Overcounting is particularly problematic when comparing rates to external sources (e.g., comparing rates of orders for a medication to a stand‐alone source of dispensing data). To avoid this issue, we limited our analyses to within‐DP comparisons and to comparisons of the same measures computed the same way across DPs. Detecting overcounting and undercounting remains a challenge, particularly when trying to validate against stand‐alone, external measures that may represent seemingly similar concepts but may be evaluated differently. In addition, we limited our initial analyses to the top 200 medications and laboratory results at each DP. While this represents just a subset of the overall data, it should still provide a sense of the magnitude of any issues. Finally, while we attempted to choose conditions and clinical concepts that capture different populations, we are limited in our generalizability in that we have findings from just 4 conditions and a set of pre‐defined clinical concepts across 3 DPs.

## 6 CONCLUSION

This project sought to develop methods to assess the harmonization and completeness of data within CDMs. While the initial investigation was limited to 3 DPs, results revealed several findings that should be explored further. When looking at data harmonization, we found that mapping errors between source data and reference terminologies may not be widespread but exist within most CDMs. Procedures to assess harmonization can help identify these mapping issues, but DPs must properly populate raw fields for that to happen. With data completeness, having access to data streams of different provenance can help identify issues, but only if there is a way to represent provenance in the CDM, and only if DPs populate those data. The potential value of looking at data by provenance has not really been raised within most research networks or network‐based learning health systems. Efforts should be made to explore its importance in future work. In the meantime, these findings can help inform study teams that are working with CDM data sources.

## FUNDING INFORMATION

This project was supported by Task Order 7540119F19002 under Master Agreement 75F40119D10037 from the US Food and Drug Administration (FDA). The FDA reviewed and approved this manuscript. Coauthors from the FDA participated in the preparation and decision to submit the manuscript for publication. The FDA had no role in data collection, management, or analysis.

## CONFLICT OF INTEREST STATEMENT

Keith Marsolo reports grants and contracts to his institution from Novartis, Amgen, Seqirus, Genentech, BMS, Bayer, and Boehringer Ingelheim. No other authors report a conflict of interest.

## Supporting information


**Table S1.** Examples of potential mapping issues from raw/ source data to reference terminologies for laboratory results and medications. The value in the code column represents a LOINC code for laboratory results and RxNorm for medications.
**Table S2.** Terminologies used within the different domains of the PCORnet® CDM.
**Table S3.** Information on data provenance for diagnoses and procedures among study DPs and for PCORnet as a whole (May 2023).
