# Supplementary material for: Assessing the harmonization of structured electronic health record data to reference terminologies and data completeness through data provenance
Source: Learn Health Syst. 2024 Oct 21;9(2):e10468. doi: 10.1002/lrh2.10468 (PMC12000768; doi:10.1002/lrh2.10468)
Supplement: Supplementary file 1 — Table S1. Examples of potential mapping issues from raw/ source data to reference terminologies for laboratory results and medications. The value in the code column represents a LOINC code for laboratory results and RxNorm for medications. Table S2. Terminologies used within the different domains of the PCORnet® CDM. Table S3. Information on data provenance for diagnoses and procedures among study DPs and for PCORnet as a whole (May 2023). [file LRH2-9-e10468-s001.docx]

**Supplementary Material**

*Supplementary Table 1: Examples of potential mapping issues from raw / source data to reference terminologies for laboratory results and medications. The value in the code column represents a LOINC code for laboratory results and RxNorm for medications.*

| **Example raw source laboratory test to be mapped:** Neutrophils (Absolute) | | |
| --- | --- | --- |
| **Code** | **Description** | **Notes** |
| 26499-4 | Neutrophils [#/volume] in Blood | Does not specify the method. Most clinical labs will use an automated counter, so should choose a more specific code. |
| 751-8 | Neutrophils [#/volume] in Blood by Automated count | Would typically be the preferred code for this test. |
| 753-4 | Neutrophils [#/volume] in Blood by Manual count | Unless it is known that the results are generated via manual count, this code would not be appropriate. |
| 754-2 | Neutrophils [#/volume] in Cerebral spinal fluid by Automated count | Specimen source is incorrect for the typical “Neutrophils (Absolute)” test, which would be blood. |
| 770-8 | Neutrophils/100 leukocytes in Blood by Automated count | This is the code for the “Neutrophils” test, which reports results as a percentage. |
| **Example raw source medication to be mapped:** fluticasone propionate RESPIRATORY (INHALATION) AEROSOL, METERED 220 ug | | |
| 896004 | 120 ACTUAT fluticasone propionate 0.22 MG/ACTUAT Metered Dose Inhaler | This is the most appropriate code for this medication, as it encapsulates the ingredient, strength and dose form. |
| 896005 | fluticasone propionate 0.22 MG/ACTUAT [Flovent] | This code specifies a brand name, and would not be appropriate unless there was knowledge that the medication was dispensed in branded form and not generic. |
| 41126 | fluticasone | Ingredient level code, but associated with two precise ingredients – fluticasone furoate and fluticasone propionate. Use of this code means it will not be possible to distinguish between medications that use the different ingredients. |
| 50121 | fluticasone propionate | Code for the precise ingredient associated with the medication, but use of this code means it will not be possible to distinguish records for the inhaler from those of a topical product with the same ingredient. |
| 895987 | fluticasone propionate 0.5 MG/ML Topical Cream | While the ingredient is the same, this code would not be correct as the strength and dose form are wrong. It is an example of how using less granular codes (e.g., ingredient-level) can lead to errors in analyses, however. Queries would not be able to distinguish between the two forms for records with ingredient-level codes. |

*Supplementary Table 2: Terminologies used within the different domains of the PCORnet® CDM.*

| **Domain** | **Supported Terminologies** | **Notes** |
| --- | --- | --- |
| Medication orders | RxNorm |  |
| Medication administrations | RxNorm NDC (National Drug Codes) | All DPs in this project used RxNorm |
| Laboratory results | LOINC (Logical Observation Identifiers Names and Codes) |  |
| Diagnoses | ICD (International Classification of Diseases) SNOMED CT (Systemized Nomenclature of Medicine – Clinical Terms) | Within PCORnet, diagnoses are primarily represented using ICD |
| Procedures | ICD  CPT (Current Procedural Terminology)  HCPCS (Healthcare Common Procedure Coding System)  LOINC  NDC  Medicare Revenue | LOINC, NDC and Revenue codes are rarely used to query procedures in PCORnet |

*Supplementary Table 3: Information on data provenance for diagnoses and procedures among study DPs and for PCORnet as a whole (May 2023).*

| **Domain** |  | **Clinician-entered only** | **EHR billing only** | **Both clinician-entered and billing** |
| --- | --- | --- | --- | --- |
| Diagnoses | DP1 |  |  | X |
|  | DP2 |  |  | X |
|  | DP3 |  | X |  |
|  | All PCORnet DPs (n=60) | 19 | 18 | 23 |
| Procedures | DP1 |  | X |  |
|  | DP2 |  |  | X |
|  | DP3 |  | X |  |
|  | All PCORnet DPs (n=60) | 16 | 20 | 24 |
